# New‐onset prediabetes/diabetes worsens overall survival in patients with cancer: A real‐world retrospective cohort study

**DOI:** 10.1111/dom.70311

**Published:** 2025-11-24

**Authors:** Maci Winn, Svenja Pauleck, Stephanie Richardson, Richard Viskochil, Prasoona Karra, Howard Colman, Jennifer A. Doherty, Yizhe Xu, Siwen Hu‐Lieskovan, Michelle Litchman, Mary C. Playdon, Sheetal Hardikar

**Affiliations:** ^1^ Department of Population Health Sciences University of Utah Salt Lake City Utah USA; ^2^ Huntsman Cancer Institute University of Utah Salt Lake City Utah USA; ^3^ Department of Exercise and Health Sciences University of Massachusetts Boston Massachusetts USA; ^4^ Department of Epidemiology Geisel School of Medicine at Dartmouth College Lebanon New Hampshire USA; ^5^ Department of Nutrition and Integrative Physiology University of Utah Salt Lake City Utah USA; ^6^ University of Utah Department of Internal Medicine Division of Epidemiology Salt Lake City Utah USA; ^7^ College of Nursing University of Utah Salt Lake City Utah USA

**Keywords:** antihyperglycemic medication, cancer, cancer survival, diabetes, prediabetes

## Abstract

**Aims:**

Cancer diagnosis may increase the risk of developing prediabetes/diabetes and subsequently worsen survival in cancer patients. However, it is unclear whether this association is influenced by the timing of hyperglycemia onset or the use of antihyperglycemic medications.

**Materials and methods:**

This study leveraged a retrospective cohort, constructed using electronic health record data, of solid tumour patients at the Huntsman Cancer Institute, a comprehensive cancer centre in Utah, USA. Adjusted Cox proportional‐hazards regression models were used to calculate hazard ratios (HR) and 95% confidence intervals (CI) to evaluate the association between new‐onset prediabetes/diabetes and overall survival.

**Results:**

Of the 7300 patients included, 23% developed prediabetes/diabetes after cancer diagnosis (mean time‐to‐onset = 2.6 years). Patients with new‐onset prediabetes/diabetes had worse overall survival compared to patients without prediabetes/diabetes [HR (95% CI): 2.97 (2.48–3.55)]. Compared to patients without prediabetes/diabetes, patients who were not prescribed antihyperglycemic medications had poorer survival than those who were prescribed antihyperglycemic medications (*n* = 665) [HR (95% CI): 2.52 (2.17–2.93) and 1.70 (1.40–2.06), respectively]. Ever use of metformin was associated with better survival [HR (95% CI): 0.32 (0.15–0.66)] compared to individuals with prescriptions of other non‐insulin antihyperglycemic medications, while ever use of insulin was associated with worse survival [HR (95% CI): 1.67 (1.16–2.42)], compared to individuals with prescription of non‐insulin antihyperglycemic medications.

**Conclusions:**

Improving clinical practice guidelines for appropriate hyperglycemia monitoring and management, especially in the first 3 years after cancer diagnosis, may improve cancer survival. Early intervention with non‐insulin antihyperglycemic medications for control of new‐onset prediabetes/diabetes may be crucial in preventing the need for insulin prescription and worsening of survival.

## 1 INTRODUCTION

Recent evidence suggests that patients who have pre‐existing prediabetes or diabetes at cancer diagnosis have worse survival compared to patients without diabetes.^1^, ^2^ Patients may also have a higher risk of developing prediabetes/diabetes after cancer diagnosis, likely resulting from a complex interplay of factors.^3^, ^4^, ^5^, ^6^, ^7^, ^8^, ^9^, ^10^ Many lifestyle changes that occur during the post‐diagnosis period, including changes in diet, smoking behaviour, and physical activity, may alter glycemic control.^8^, ^11^, ^12^, ^13^ Cancer treatments can also contribute to the development of prediabetes/diabetes, with chemotherapy, corticosteroids, and other therapies potentially impairing glucose metabolism and increasing the risk of hyperglycemia post‐cancer diagnosis. A higher risk of new‐onset prediabetes/diabetes in patients with cancer has been observed in several large cohorts, including the Women's Health Initiative, National Health Interview Survey, National Health Insurance Service–National Sample Cohort, and Surveillance, Epidemiology, and End Results (SEER)‐Medicare database.^3^, ^4^, ^5^, ^6^, ^7^, ^8^, ^9^ This increased risk of developing prediabetes/diabetes puts cancer patients at a higher risk for cardiovascular diseases, other cancers, and worse overall survival.^3^, ^8^, ^14^, ^15^ Consequently, effective management of hyperglycemia post‐cancer diagnosis may be critical in improving cancer survival.

Existing studies have not yet evaluated the timing of prediabetes/diabetes onset or accounted for the treatment of such new‐onset prediabetes/diabetes after cancer diagnosis in relation to cancer survival, which is essential to better inform clinical practice. The aim of this study was to evaluate the association of new‐onset prediabetes/diabetes after cancer diagnosis with overall survival, accounting for the time interval between cancer and prediabetes/diabetes diagnosis and antihyperglycemic medication use during the post‐diagnosis period, using real‐world electronic health record (EHR) data on patients with solid cancers.

## 2 METHODS

### 2.1 Patient population

All adult patients (≥18 years) enrolled in the Total Cancer Care (TCC) study at the Huntsman Cancer Institute, a comprehensive cancer care centre in the University of Utah Health system in the United States, with a primary solid cancer diagnosis between 2014 and 2021 (*n* = 10 091) were eligible for inclusion. The follow‐up period for all patients ended either at the date of death or, for those still alive, at the date of the final data extraction in 2021. The cohort was restricted to individuals with a first primary solid cancer diagnosis; patients with secondary cancers were excluded. Clinical, demographic, and treatment data were extracted using biomedical informatics techniques and clinical text mining. Patients with a prediabetes or diabetes diagnosis, or those prescribed antihyperglycemic medications prior to or at the time of cancer diagnosis, were excluded. To reduce misclassification, we also excluded patients who were prescribed antihyperglycemic medications without a corresponding diagnosis of prediabetes or diabetes. Additionally, patients with hematologic cancers were also excluded, owing to the high prevalence of corticosteroid use as a primary cancer treatment (often resulting in subsequent hyperglycemia) (Figure S1).^16^

### 2.2 Exposures

The diagnosis of new‐onset diabetes/prediabetes and the use of antihyperglycemic medications were exposures for our analyses. Patients with new‐onset diabetes or prediabetes were identified using ICD‐9 and ICD‐10 diabetes diagnosis codes (Table S1, Supporting Information), or through prescriptions of antihyperglycemic medications, with ICD code date or prescription dates serving as the identification point, respectively. Antihyperglycemic medication (metformin, sulfonylureas, thiazolidinediones, glucagon‐like peptide‐1 [GLP‐1] agonists, dipeptidyl peptidase‐4 [DPP‐4] inhibitors, sodium‐glucose cotransporter‐2 [SGLT‐2] inhibitors, and insulin) use was determined based on prescription dates in the EHR. For subgroup analysis by diabetes type, only patients who had a type 1 diabetes ICD code and were prescribed insulin were classified as having type 1 diabetes.^17^

### 2.3 Outcomes

The primary outcome for all analyses was overall survival, defined as death from any cause. Vital status was determined by the documented date of death in the EHR.

### 2.4 Covariates

Models were adjusted for age, sex, race (White/non‐White), BMI (<18.5, ≥18.5, ≥25, <30, ≥30 kg/m^2^), smoking status (never/passive/former/current), corticosteroid use (before or at cancer diagnosis/after cancer diagnosis/before and after cancer diagnosis), cancer stage, cancer treatment (no treatment/surgery only/systemic treatment only/other), obesity‐related cancer (no/yes), and comorbidity status (cardiovascular disease, chronic obstructive pulmonary disease, or chronic kidney disease at cancer diagnosis; no/yes; Table S1).^18^ If BMI at cancer diagnosis was missing, it was calculated using height and weight measurements closest to cancer diagnosis. Improbable BMI values (>200 or <12 kg/m^2^) were reclassified as missing. Obesity‐related cancers were defined using ICD codes and tumour histology, as outlined by the International Agency for Research on Cancer (postmenopausal breast [limited to ≥50 years of age as menopausal status was unavailable], colorectal, ovarian, endometrial, renal, thyroid, meningioma, gallbladder, gastric cardia, oesophageal adenocarcinoma, liver, and pancreatic cancers [excluding multiple myeloma due to receipt of corticosteroids as primary treatment]).^19^, ^20^

### 2.5 Statistical analysis

Descriptive statistics were presented as numbers and percentages for categorical variables and as means and standard deviations for continuous variables, computed by new‐onset prediabetes/diabetes status. For all analyses, patients were included in the cohort at the time of their cancer diagnosis. Survival outcomes were then assessed using adjusted Cox proportional‐hazards regression models, which calculated hazard ratios (HR) and 95% confidence intervals (CI). Covariates with missing values were included as a separate missing category to avoid the exclusion of patients from the regression models. For primary analyses, all prediabetes/diabetes diagnoses were included. We performed additional stratified analyses by diabetes type (prediabetes, type 1 diabetes, and type 2 diabetes) and antihyperglycemic treatment status (treated/untreated). Lastly, to investigate whether the timing of diabetes onset after cancer diagnosis influenced the results, we categorised patients into different time intervals based on the time of prediabetes/diabetes development. These intervals were defined as 0–1 year, >1–3 years, and ≥3 years after cancer diagnosis, with “time zero” corresponding to 0, 1, and 3 years from the date of cancer diagnosis for each respective interval.

To account for immortal time bias, new‐onset prediabetes/diabetes was treated as a time‐dependent exposure. As obesity shares many common pathways with both diabetes and cancer (including inflammation, gut microbiome alterations, cytokine and hormone release, dyslipidaemia, insulin resistance, and hyperglycemia^21^), we performed a subgroup analysis by obesity‐related cancer diagnosis. We also performed subgroup analyses by BMI group, sex, and cancer stage. We performed a set of sensitivity analyses, including (i) a complete case analysis excluding all observations with missing covariates, (ii) removing patients with pancreatic cancer due to the high likelihood (~80%) of glucose intolerance among these patients, and (iii) accounting for undiagnosed hyperglycemia at cancer diagnosis by allowing a 6‐month lag period for hyperglycemia development.^22^

Lastly, we evaluated the association between antihyperglycemic medication class and overall survival by (i) comparing the use of each antihyperglycemic medication class (as time‐dependent exposure) to patients without new‐onset prediabetes/diabetes and (ii) comparing the use of each antihyperglycemic medication class to the use of other antihyperglycemic medications (ever use of a single non‐insulin antihyperglycemic medication class vs. other non‐insulin medications, and ever use of insulin vs. non‐insulin antihyperglycemic medications) with medication treated as a time‐fixed covariate.

All statistical analyses were performed using R (version 4.3.2, R Foundation for Statistical Computing, Vienna, Austria), and significance was determined at *α* = 0.05.

## 3 RESULTS

Descriptive statistics overall and by new‐onset diabetes status are outlined in Table 1, and the specific cancer types represented in the cohort are listed in Table S2. To support comparison and aid interpretation, baseline characteristics and outcomes for patients excluded due to pre‐existing diabetes are provided in Table S3. Overall, a total of 7300 patients were included in the analysis, of whom 23% (1645) developed prediabetes or diabetes after cancer diagnosis. A higher proportion of patients who developed prediabetes/diabetes after cancer diagnosis used corticosteroids (50% vs. 36%), had obesity (41% vs. 27%), and died (22% vs. 13%) than patients without prediabetes/diabetes.

**TABLE 1:** Descriptive characteristics of the study population by new‐onset prediabetes/diabetes status (*N* = 7300).

New‐onset diabetes	Yes (*N* = 1645)	No (*N* = 5655)	Total (*N* = 7300)
	*N* (%) or mean (SD)	*N* (%) or mean (SD)	*N* (%) or mean (SD)
Age (years)	58.0 (13.9)	56.5 (14.7)	56.9 (14.5)
Sex			
Female	818 (49.7)	2937 (51.9)	3755 (51.4)
Male	827 (50.3)	2718 (48.1)	3545 (48.6)
Race			
White	1492 (90.7)	5279 (93.4)	6771 (92.8)
Black	8 (0.5)	32 (0.6)	40 (0.5)
Asian	22 (1.3)	64 (1.1)	86 (1.2)
Native Hawaiian/PI	15 (0.9)	22 (0.4)	37 (0.5)
American Indian/Alaska Native	24 (1.5)	53 (0.9)	77 (1.1)
Multi‐racial	12 (0.7)	28 (0.5)	40 (0.5)
Missing	72 (4.4)	177 (3.1)	249 (3.4)
Cancer stage			
Stage 0	53 (3.2)	190 (3.4)	243 (3.3)
Stage I	375 (22.8)	1606 (28.4)	1981 (27.1)
Stage III	287 (17.4)	1010 (17.9)	1297 (17.8)
Stage III	250 (15.2)	925 (16.4)	1175 (16.1)
Stage IV	283 (17.2)	813 (14.4)	1096 (15.0)
Missing	397 (24.1)	1111 (19.6)	1508 (20.7)
Cancer treatment			
No treatment	46 (2.8)	136 (2.4)	182 (2.5)
Surgery only	470 (28.6)	1998 (35.3)	2468 (33.8)
Systemic treatment only	91 (5.5)	293 (5.2)	384 (5.3)
Other	1038 (63.1)	3228 (57.1)	4266 (58.4)
Corticosteroid use			
None	827 (50.3)	3598 (63.6)	4425 (60.6)
Before/at cancer diagnosis	30 (1.8)	149 (2.6)	179 (2.5)
After cancer diagnosis	731 (44.4)	1722 (30.5)	2453 (33.6)
Before and after cancer diagnosis	57 (3.5)	186 (3.3)	243 (3.3)
Obesity‐related cancer	633 (38.5)	1958 (34.6)	2591 (35.5)
BMI (kg/m^2^)			
Normal weight	285 (17.3)	1614 (28.5)	1899 (26.0)
Underweight	18 (1.1)	101 (1.8)	119 (1.6)
Overweight	473 (28.8)	1890 (33.4)	2363 (32.4)
Obese	680 (41.3)	1514 (26.8)	2194 (30.1)
Missing	189 (11.5)	536 (9.5)	725 (9.9)
Smoking			
Never	199 (12.1)	632 (11.2)	831 (11.4)
Passive	3 (0.2)	7 (0.1)	10 (0.1)
Quit	29 (1.8)	124 (2.2)	153 (2.1)
Yes	31 (1.9)	106 (1.9)	137 (1.9)
Missing	1383 (84.1)	4786 (84.6)	6169 (84.5)
Vital status			
Alive	1281 (77.9)	4949 (87.5)	6230 (85.3)
Died	364 (22.1)	706 (12.5)	1070 (14.7)

Abbreviations: BMI, body mass index (closest to cancer diagnosis); PI, Pacific Islander.

Among patients who developed prediabetes/diabetes, 670 (41%) had prediabetes, 928 (56%) had type 2 diabetes, and 33 (2%) had type 1 diabetes. The mean time to onset of prediabetes/diabetes was 2.6 years (SD = 4.5 years), with most (58%) developing diabetes within the first year of cancer diagnosis. More than half of the patients who developed prediabetes/diabetes during the post‐diagnosis period were not prescribed antihyperglycemic medications (60%). Among those treated with antihyperglycemic medications, the most common medications prescribed were metformin (80%) and insulin (35%).

### 3.1 New‐onset prediabetes/diabetes and survival

During ~31 836 person‐years of follow‐up (range: 0.02–42.3 years) for the TCC DiMe cohort, there were 1070 (15%) deaths. Patients who developed prediabetes/diabetes after cancer diagnosis had a nearly twofold worse overall survival compared to patients who did not [HR (95% CI): 1.99 (1.77–2.24)] (Table 2). The strongest relationship was observed with prediabetes [HR (95% CI): 2.97 (2.48–3.55)], but the magnitude of association for type 1 diabetes was comparable [HR (95% CI): 2.71 (1.21–6.07)], and significantly elevated for type 2 diabetes [HR (95% CI): 1.71 (1.45–2.01)]. New‐onset prediabetes/diabetes was most strongly associated with overall survival in the first 3 years after cancer diagnosis [HR (95% CI): 0–1 year 1.73 (1.46–2.06); 1–3 years 2.24 (1.73–2.90); ≥3 years 1.18 (0.90–1.54)]. Compared to patients without prediabetes/diabetes, patients who were not prescribed antihyperglycemic medications had worse overall survival than those who were (*n* = 665) [HR (95% CI): 2.52 (2.17–2.93) and 1.70 (1.40–2.06), respectively].

**TABLE 2:** Adjusted hazard ratios and 95% confidence intervals for the association of new‐onset prediabetes/diabetes treated as a time‐varying exposure with overall survival compared to patients that did not develop hyperglycemia after cancer diagnosis (*N* = 7300).

	*N* (%)	HR (95% CI)
Incident prediabetes/diabetes		
No	5655 (77.5)	REF
Yes	1645 (22.2)	1.99 (1.77–2.24)
By prediabetes/diabetes type		
No prediabetes/diabetes	5655 (77.5)	REF
Prediabetes	670 (9.1)	2.97 (2.48–3.55)
Type 2	928 (12.5)	1.71 (1.45–2.01)
Type 1	33 (0.4)	2.71 (1.21–6.07)
Other	14 (0.2)	1.18 (0.26–5.31)
By diabetes treatment status		
No prediabetes/diabetes	5655 (77.5)	REF
Treated prediabetes/diabetes	665 (9.0)	1.70 (1.40–2.06)
Untreated prediabetes/diabetes	980 (13.2)	2.52 (2.17–2.93)
By time to prediabetes/diabetes onset		
No prediabetes/diabetes	5655 (77.5)	REF
0–1 years after cancer diagnosis	949 (12.8)	1.73 (1.46–2.06)
1–3 years after cancer diagnosis	299 (4.0)	2.24 (1.73–2.90)
≥3 years after cancer diagnosis	397 (5.4)	1.18 (0.90–1.54)

*Note*: All models were adjusted for age, sex, race, cancer stage, cancer treatment type, corticosteroid use, smoking, body mass index (BMI) at cancer diagnosis, and presence of comorbidities. The “other” category for diabetes type includes electronic health record diagnoses not otherwise classified as prediabetes, type 1, or type 2 diabetes, and includes drug‐induced, gestational, and unspecified diabetes.

**FIGURE 1:**
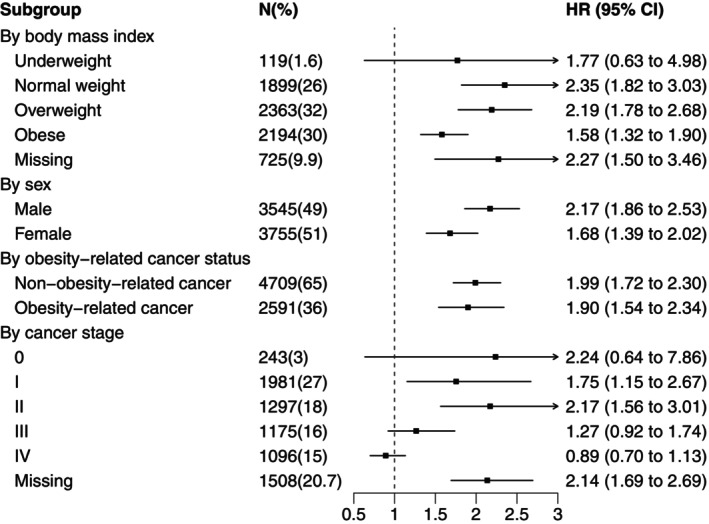
Forest plot subgroup analyses of new‐onset prediabetes/diabetes after cancer diagnosis and overall survival (*N* = 7300). The normal weight body mass index (BMI) group excludes underweight patients (BMI < 18 kg/m^2^). All models were adjusted for age, sex, race, cancer stage, cancer treatment type, corticosteroid use, smoking, BMI at cancer diagnosis, and presence of comorbidities.

In subgroup analyses, new‐onset prediabetes/diabetes was associated with worse overall survival among all BMI groups, with the strongest associations observed in normal‐weight and overweight patients [HR (95% CI): underweight 1.77 (0.63–4.98); normal weight 2.35 (1.82–3.03); overweight 2.19 (1.78–2.68); obese 1.58 (1.32–1.90)] (Figure 1). New‐onset prediabetes/diabetes was also associated with worse survival in both obesity‐related and non‐obesity‐related cancers [HR (95% CI): 1.90 (1.54–2.34), 1.99 (1.72–2.30), respectively], and in both sexes [HR (95% CI): male 2.17 (1.86–2.53), female 1.68 (1.39–2.02)].

In lag analysis, excluding patients who developed prediabetes/diabetes in the first 6 months after cancer diagnosis, a threefold elevated risk of death was observed among patients with a new‐onset prediabetes/diabetes compared to patients without prediabetes/diabetes [HR (95% CI): 3.06 (2.61–3.60)] (Table S4). Using only those with complete data on confounders or excluding pancreatic cancer cases did not change the magnitude or direction of the results (Table S4).

### 3.2 Antihyperglycemic medication use and survival

Ever use of metformin, insulin, sulfonylureas, DPP‐4 inhibitors, and SGLT‐2 inhibitors was associated with worse overall survival compared to patients who did not develop prediabetes/diabetes (Table 3). Conversely, ever use of GLP‐1 agonists was associated with better survival [HR (95% CI): 0.64 (0.26–1.54)] compared to patients without new‐onset prediabetes/diabetes, though this result was not statistically significant. Among patients prescribed non‐insulin antihyperglycemic medications (*N* = 595), ever use of metformin was significantly associated with better overall survival compared to the prescription of other non‐insulin agents [HR (95% CI): 0.32 (0.15–0.66)] (Table 4). The use of insulin was associated with worse overall survival alone [HR (95% CI): 2.95 (1.74–5.01)] and in combination with non‐insulin antihyperglycemic medications [HR (95% CI): 1.32 (0.88–1.98)] compared to those who were prescribed only non‐insulin antihyperglycemic medications, though only the association with insulin alone was significant (Table 4).

**TABLE 3:** Adjusted hazard ratios and 95% confidence intervals for the association of ever use of antihyperglycemic medications with overall survival compared to patients without a prediabetes/diabetes diagnosis (*N* = 7300).

Medication class	*N* (%)	HR (95% CI)
Treatment by any medication		
No prediabetes/diabetes	5655 (77.5)	REF
Ever	665 (9.0)	1.7 (1.40–2.06)
Metformin		
No prediabetes/diabetes	5655 (77.5)	REF
Ever	530 (8.4)	1.60 (1.28–2.00)
Sulfonylureas		
No prediabetes/diabetes	5655 (77.5)	REF
Ever	146 (2.3)	1.98 (1.39–2.82)
DPP‐4 inhibitors		
No prediabetes/diabetes	5655 (77.5)	REF
Ever	90 (1.4)	1.32 (0.73–2.40)
GLP‐1 agonists		
No prediabetes/diabetes	5655 (77.5)	REF
Ever	78 (1.1)	0.64 (0.26–1.54)
SGLT‐2 inhibitors		
No prediabetes/diabetes	5655 (77.5)	REF
Ever	54 (0.9)	1.42 (0.66–3.04)
Thiazolidinediones		
No prediabetes/diabetes	5655 (77.5)	REF
Ever	27 (0.4)	0.98 (0.31–3.12)
Insulin vs. non‐insulin medications^a^		
No prediabetes/diabetes	5655 (77.5)	REF
Non‐insulin antihyperglycemics only	430 (5.9)	0.97 (0.75–1.28)
Non‐insulin antihyperglycemics + insulin	165 (2.3)	1.28 (0.91–1.80)
Insulin only	70 (1.0)	2.89 (1.89–4.43)

*Note*: All models were adjusted for age, sex, race, cancer stage, cancer treatment type, corticosteroid use, smoking, body mass index (BMI) at cancer diagnosis, and presence of comorbidities. Patients with untreated prediabetes/diabetes or those prescribed medications other than the one of interest were excluded from each individual medication analysis.

Abbreviations: DPP‐4, dipeptidyl peptidase‐4; GLP‐1, glucagon‐like peptide‐1; SGLT‐2, sodium‐glucose cotransporter‐2.

^a^ Patients with untreated prediabetes/diabetes were excluded from analyses (*N* = 980).

**TABLE 4:** Adjusted hazard ratios and 95% confidence intervals for the association of ever use of antihyperglycemic medications with overall survival among all patients treated with antihyperglycemic medications.

Non‐insulin antihyperglycemic medication class^a^	*N* (%)	HR (95% CI)
Metformin		
Never	37 (9)	REF
Ever	393 (91)	0.32 (0.15–0.66)
Sulfonylureas		
Never	349 (81)	REF
Ever	81 (19)	0.93 (0.46–1.87)
DPP‐4 inhibitors		
Never	372 (87)	REF
Ever	58 (13)	1.12 (0.53–2.35)
GLP‐1 agonists		
Never	395 (92)	REF
Ever	35 (8)	0.42 (0.09–1.96)
SGLT‐2 inhibitors		
Never	401 (93)	REF
Ever	29 (7)	0.95 (0.26–3.42)
Thiazolidinediones		
Never	412 (96)	REF
Ever	18 (4)	0.31 (0.04–2.15)

Insulin^b^	*N* (%)	HR (95% CI)
Non‐insulin antihyperglycemics only	430 (65)	REF
Non‐insulin antihyperglycemics + insulin	165 (25)	1.32 (0.88–1.98)
Insulin only	70 (11)	2.95 (1.74–5.01)

*Note*: All models were adjusted for age, sex, race, cancer stage, cancer treatment type, corticosteroid use, smoking, body mass index (BMI) at cancer diagnosis, and presence of comorbidities.

Abbreviations: DPP‐4, dipeptidyl peptidase‐4; GLP‐1, glucagon‐like peptide‐1; SGLT‐2, sodium‐glucose cotransporter‐2.

^a^ Ever use of each non‐insulin antihyperglycemic medication class compared to the use of other non‐insulin antihyperglycemic agents (*N* = 430 patients treated with non‐insulin antihyperglycemic medication only).

^b^ Ever use of insulin compared to the use of non‐insulin antihyperglycemic agents.

## 4 DISCUSSION

Using a real‐world cohort of patients with cancer, we observed that new‐onset prediabetes/diabetes after cancer diagnosis was associated with a nearly twofold worse overall survival compared to patients without a prediabetes/diabetes diagnosis. Emerging evidence suggests that cancer diagnosis and subsequent cancer treatment may have direct effects on glucose control, thereby increasing the risk of developing diabetes after cancer diagnosis.^3^, ^4^, ^5^, ^6^, ^7^, ^8^, ^9^, ^10^ The exact mechanisms contributing to this observation are not completely understood. However, diabetes and cancer share many molecular pathways and behavioral risk factors, all of which may contribute to a bidirectional relationship between metabolic dysfunction and cancer.

The increased risk of prediabetes/diabetes may stem from the direct effects of the tumour environment. Insulin resistance may impact tumour growth and result in the decreased production of sex‐hormone‐binding globulin, which is implicated in breast and endometrial cancers.^21^, ^23^ Inflammatory responses triggered by cancer could contribute to the onset of insulin resistance or worsen cancer cachexia.^24^ Lifestyle changes, including changes in diet and appetite, sleep patterns, smoking habits, physical activity, sedentary behaviour, and increased stress, can also contribute to altered glycemic control.^8^, ^11^, ^12^, ^13^, ^25^, ^26^, ^27^ Improved weight management after cancer diagnosis may decrease the risk of hyperglycemia and subsequent worsening of survival.^28^ In addition to cancer progression, hyperglycemia and overt diabetes may also contribute to a higher risk of cardiovascular disease‐related deaths, further worsening overall survival in patients with cancer.^29^

Few studies have examined the association of prediabetes with cancer survival. However, current evidence suggests that hyperglycemia, even below the level warranting a clinical diabetes diagnosis, may worsen cancer survival.^30^ We observed a threefold worse survival in patients with new‐onset prediabetes compared to patients without prediabetes/diabetes. Thus, monitoring and treating patients for even slight elevations in glucose may impact overall survival in cancer patients, particularly in the first few years after cancer diagnosis.^3^, ^8^

In our study, the association between new‐onset prediabetes/diabetes and survival was slightly stronger in males than in females. This may be due to cancer type differences by sex, as patients with prostate cancer receive androgen deprivation therapy, which is known to induce hyperglycemia.^31^ We also observed worse survival in patients with non‐metastatic cancer (stages I–III). Most studies did not examine or observe differences in the association between new‐onset diabetes and survival by cancer stage.^3^, ^4^, ^6^, ^7^ Li et al. observed both an increasing trend of glucose intolerance by tumour stage and worse survival among patients with metastatic cancer.^5^ However, this study was conducted only in patients with pancreatic cancer, which may account for the differences from our study, as most late‐stage pancreatic cancers will result in glucose intolerance.^22^ Diabetes risk may also disproportionately impact certain population groups. Hashibe et al. observed a notably higher risk of developing type 2 diabetes among Asian, Native Hawaiian, and Pacific Islander lung cancer survivors compared to non‐Hispanic white survivors. Further research in large, diverse cohorts is essential to fully understand these disparities and their broader implications.^9^

We observed a potential obesity paradox where patients with new‐onset prediabetes/diabetes after cancer diagnosis and who were overweight or obese had better survival compared to patients with BMI in the normal weight range. This obesity paradox has also been observed in numerous studies.^32^, ^33^, ^34^ The exact mechanisms behind this paradox are unclear, but may be explained by altered gene expression, protection against cancer cachexia, and improved cancer treatment efficacy.^32^ Cancers attributable to obesity may also be less aggressive, potentially explaining the better survival and differences observed by cancer stage.^32^, ^35^ Further research is needed to understand the clinical relevance of these observations.

Recent evidence also suggests that the use of oral antihyperglycemic medications, like metformin, DPP‐4 inhibitors, and SGLT‐2 inhibitors, may improve overall and cancer‐specific survival, while sulfonylureas have often been associated with poorer survival.^36^, ^37^, ^38^, ^39^, ^40^, ^41^, ^42^, ^43^, ^44^, ^45^ Overall, the survival benefit of metformin has been well documented in patients with cancer and may be due to a reduction in tumour cell growth and proliferation.^36^, ^46^ Currie et al. observed reduced cancer mortality with metformin use, even when compared to individuals without diabetes, suggesting a potential broader impact on both cancer prevention and treatment.^47^ The limited studies on the association between GLP‐1 agonists and cancer survival have reported better overall survival, reduced major adverse cardiovascular events, and protection from chemotherapy‐induced cardiotoxicity.^48^, ^49^, ^50^ Future studies powered to evaluate individual antihyperglycemic medications and medications by cancer site are still needed to fully understand these associations, especially considering the recent rapid increases in non‐insulin glucose‐lowering medication use.^51^

The use of exogenous insulin may increase the risk of cancer‐related death due to its mitogenic properties.^36^, ^37^, ^47^ Insulin and insulin‐like growth factor signalling may directly promote tumour growth and progression through tumour cell‐specific mechanisms, including increased cell division, glucose metabolism, and epithelial‐to‐mesenchymal transition.^52^ In our study, compared to patients treated with non‐insulin antihyperglycemic medications, insulin use, particularly as monotherapy, was associated with poorer overall survival. This association may reflect treatment‐specific effects, including the potential mitogenic properties of insulin, but could also reflect the severity of underlying metabolic dysfunction in patients with more advanced or difficult‐to‐control diabetes, which necessitates the use of insulin therapy. Controlling glucose levels using non‐insulin antihyperglycemic medications rather than insulin in patients with cancer may prevent diabetes progression and the subsequent need for insulin administration.^53^

Many cancer treatments also contribute to diabetes development. Fluorouracil‐based chemotherapies induce diabetes by causing acute and chronic damage to pancreatic beta cells.^54^ Corticosteroids, commonly used for anti‐tumour effects or symptom management, increase insulin resistance, leading to drug‐induced hyperglycemia that can persist and eventually result in diabetes.^55^ Endocrine therapies, like tamoxifen, aromatase inhibitors, and GnRH agonists, increase diabetes risk potentially by reducing insulin sensitivity and promoting apoptosis in pancreatic β‐cells.^31^, ^56^, ^57^ Additionally, patients undergoing thyroidectomy or orchiectomy for thyroid or prostate cancer face an elevated diabetes risk due to disruptions in glucose metabolism if not properly managed with hormone replacement.^57^, ^58^ Radiation therapy has also been linked to an increased incidence of diabetes in cancer survivors. Emerging therapies, such as immune checkpoint inhibitors, can induce hyperglycemia or even type 1 diabetes by triggering pancreatic beta‐cell destruction, which may lead to treatment discontinuation and worsen survival outcomes.^59^, ^60^, ^61^, ^62^ Furthermore, patients with diabetes receiving anthracycline‐based chemotherapy are at increased risk of severe cardiovascular events, including heart failure and cardiovascular death.^63^ This suggests that diabetes may not only be potentially driving cancer progression but also raising the risk of treatment‐related mortality. In the current study, new‐onset prediabetes/diabetes was most strongly associated with survival within the first 3 years of cancer diagnosis, further underlying the potential role of cancer treatments in the development of hyperglycemia. Due to limited cancer treatment‐related data for our analyses, we did not focus on cancer treatments and their associations with new‐onset prediabetes/diabetes. However, further research is needed to explore the impact of emerging therapies on diabetes risk.

Despite the emerging evidence, there are currently no standardised guidelines for monitoring hyperglycemia following a cancer diagnosis.^12^, ^64^, ^65^, ^66^, ^67^ Individuals with cancer often have more comorbidities, potentially reducing primary care utilisation and antihyperglycemic medication adherence, thus exacerbating glucose intolerance.^68^, ^69^, ^70^ This phenomenon has been well‐documented in patients with pre‐existing or prevalent diabetes. These patients have also been shown to receive less diabetes care after cancer diagnosis and are at a higher risk of diabetes‐related complications and death.^71^, ^72^ Thus, routine and proactive hyperglycemia monitoring in patients with cancer may be crucial in the timely identification and management of new or worsening glucose intolerance. Additionally, given the potential adverse impact of insulin on cancer survival outcomes, there is a compelling rationale for early intervention with non‐insulin antihyperglycemic agents and the integration of endocrinology support within oncology care.^73^, ^74^, ^75^ Rigorous, targeted research is essential to optimise glycemic management in cancer patients to improve both cancer‐related and metabolic outcomes.

This study has some limitations. As this study was conducted using EHR data, we were unable to obtain information on potentially relevant confounders, including physical activity, diet, and alcohol use. Additionally, some covariates had missing data, particularly smoking, BMI, and cancer stage. However, we conducted a complete case analysis to account for missingness, and the results did not differ. Our statistical power was insufficient for examining associations by cancer and diabetes type (particularly type 1). Given the heterogeneity of cancer types in terms of clinical and treatment characteristics, future research involving large, diverse cohorts of cancer patients is essential. While our analysis accounted for broad categories of cancer treatments, detailed data on specific treatment regimens were not available, limiting our ability to fully assess their impact on survival. As this study utilised EHR data, traditional loss to follow‐up could not be systematically assessed. We acknowledge that some patients may not have returned for subsequent visits due to death or clinical deterioration, which may not have been consistently captured in the records and represents a limitation of EHR‐based analyses. Structured biomarker data, including HbA1c and fasting glucose, were not available in this dataset. Thus, prediabetes/diabetes diagnoses were determined using ICD codes alone, which could lead to some misclassification if an individual had hyperglycemia at or above clinical reference ranges but had not received a formal diagnosis. However, identifying all patients with hyperglycemia using more comprehensive measurements would potentially make these associations stronger and would bias our results toward the null. We were unable to assess adherence to antihyperglycemic medications, duration of use, or care setting, limiting our ability to distinguish between transient and sustained therapy and highlighting the need for future research. Further, we were unable to ascertain the specific indications for antihyperglycemic prescriptions, which may have led to potential misclassification, as some medications are prescribed for conditions such as obesity, renal protection, or cardiovascular risk reduction. To minimise this risk, however, we excluded patients who were prescribed antihyperglycemic medications without a documented diagnosis of prediabetes or diabetes, as noted above. Lastly, we were unable to evaluate cancer‐specific survival, and it is possible that causes of death other than cancer contributed to overall survival. However, we attempted to control for major comorbidities by adjusting for them in our models.

This study has many strengths. Despite the limitations of EHRs, our data represent real‐world clinical populations and may be more generalisable compared to cohort and clinical trial populations at risk of healthy user bias. Further, we were able to capture long‐term data, including cancer characteristics, comorbidities like cardiovascular disease, and overall survival, as well as a detailed clinical history prior to cancer diagnosis in a large cohort of patients. Currently, very few studies have examined the association between new‐onset diabetes after cancer diagnosis and survival.^3^ In this study, we performed time‐varying analyses for new‐onset prediabetes/diabetes and stratified by time to prediabetes/diabetes development that may represent different risk periods owing to different cancer and other treatment, lifestyle, and tumour stages. Including medication prescriptions in addition to ICD diagnosis codes increases the sensitivity of identifying patients with prediabetes/diabetes compared with diagnosis codes alone. We also identified prediabetes in addition to diabetes diagnoses and accounted for a variety of antihyperglycemic medications, adding to the growing literature on new‐onset prediabetes/diabetes and survival in patients with cancer.

## 5 CONCLUSIONS

Improving clinical practice guidelines for appropriate hyperglycemia monitoring and management, especially in the first 3 years after cancer diagnosis, may improve cancer survival. Furthermore, early intervention with non‐insulin antihyperglycemic medications for control of new‐onset prediabetes/diabetes may be crucial in preventing the need for insulin prescription and worsening of survival. More studies in large, prospective cohorts with comprehensive biomarker assessments (HbA1c, C‐peptide) accounting for complex cancer treatments and cancer‐related deaths are needed to better understand the relationship between prediabetes/diabetes diagnosed after a cancer diagnosis and subsequent survival. In future studies, we also aim to incorporate time‐varying exposures to better capture changes in antihyperglycemic treatment over time, including medication class switching, dose escalation, and adherence patterns. This may allow for a more detailed understanding of how dynamic treatment strategies can influence survival outcomes.

## AUTHOR CONTRIBUTIONS

All authors contributed to writing and editing the manuscript. Maci Winn, Sheetal Hardikar, Prasoona Karra, and Mary C. Playdon contributed to the design of the manuscript. Maci Winn, Sheetal Hardikar, Svenja Pauleck, Prasoona Karra, Richard Viskochil, and Stephanie Richardson contributed to the data analysis and conduct/collection.

## CONFLICT OF INTEREST STATEMENT

The authors declare no conflicts of interest.

## ETHICS STATEMENT

Informed consent was obtained from all study participants by the original data collectors. Access to these data is highly regulated, as these data include identifiable information. Authorization for analyses was given through the Institutional Review Board (IRB).

## Supporting information


**Data S1.** Supporting Information tables.

**Figure S1.** Flow diagram of inclusion and exclusion criteria.

## Data Availability

The data underlying this article cannot be shared due to the privacy of individuals who participated in the study. Additional summary‐level data without individual data and all analysis code is available upon request.
