# Supplementary material for: New‐onset prediabetes/diabetes worsens overall survival in patients with cancer: A real‐world retrospective cohort study
Source: Diabetes Obes Metab. 2025 Nov 24;28(2):1247–57. doi: 10.1111/dom.70311 (PMC12649824; doi:10.1111/dom.70311)
Supplement: Supplementary file 1 — Data S1. Supporting Information tables. [file DOM-28-1247-s001.docx]

**SUPPLEMENTARY TABLES**

**Supplementary Table 1:** ICD-9 and ICD-10 codes used to identify prediabetes, diabetes, and comorbidity diagnoses.

| **Prediabetes/diabetes diagnoses** | **ICD Code(s)** |
| --- | --- |
| Hyperglycemia/prediabetes | R73- |
| Type 2 diabetes | E11-, 250.x0, 250.x2, and 250.x4 |
| Type 1 diabetes | E10-, 250.x1, 250.x3, and 250.x5 |
| diabetes after underlying conditions | E08- |
| Drug induced diabetes | E09- |
| Other diabetes | E13- |
| **Comorbidity diagnoses** | **ICD Code(s)** |
| Cerebral infarction | 434- and I63- |
| Myocardial infarction | 410-, 21-, and I22- |
| Cardiac arrest | 427.5 and I46- |
| Chronic ischemic heart disease | 414- and I25- |
| Heart failure | 428- and I50- |
| Pulmonary embolism | 415.19 and I26- |
| Peripheral vascular disease | 443.9 and I73.9 |
| Atherosclerosis | 440- and I70- |
| Other venous embolism and thrombosis | 453- and I82- |
| Chronic obstructive pulmonary disease | 493.2 and J44- |
| Chronic kidney disease and hypertensive kidney disease | 585-, 403-, N18-, I12-, and I13- |

**Supplementary Table 2:** Solid tumor diagnoses among patients with cancer, stratified by new-onset prediabetes/diabetes status (N = 7,300).

| **New-onset diabetes** | **Yes (N=1645)** | **No (N=5655)** | **Total (N=7300)** |
| --- | --- | --- | --- |
| **Cancer site** | ***N (%)*** | ***N (%)*** | ***N (%)*** |
| Breast | 204 (12.4%) | 1013 (17.9%) | 1217 (16.7%) |
| Prostate | 170 (10.3%) | 859 (15.2%) | 1029 (14.1%) |
| Skin | 117 (7.1%) | 627 (11.1%) | 744 (10.2%) |
| Nervous System | 189 (11.5%) | 428 (7.6%) | 617 (8.5%) |
| Lung | 121 (7.4%) | 349 (6.2%) | 470 (6.4%) |
| Bladder | 92 (5.6%) | 228 (4.0%) | 320 (4.4%) |
| Uterine | 86 (5.2%) | 203 (3.6%) | 289 (4.0%) |
| Pancreatic | 84 (5.1%) | 177 (3.1%) | 261 (3.6%) |
| Renal | 61 (3.7%) | 191 (3.4%) | 252 (3.5%) |
| Thyroid | 72 (4.4%) | 166 (2.9%) | 238 (3.3%) |
| Oral | 65 (4.0%) | 161 (2.8%) | 226 (3.1%) |
| Colon | 50 (3.0%) | 174 (3.1%) | 224 (3.1%) |
| Ovarian | 36 (2.2%) | 139 (2.5%) | 175 (2.4%) |
| Connective Tissue | 44 (2.7%) | 114 (2.0%) | 158 (2.2%) |
| Rectal | 28 (1.7%) | 108 (1.9%) | 136 (1.9%) |
| Endocrine | 44 (2.7%) | 78 (1.4%) | 122 (1.7%) |
| Musculoskeletal | 20 (1.2%) | 77 (1.4%) | 97 (1.3%) |
| Head and Neck | 19 (1.2%) | 68 (1.2%) | 87 (1.2%) |
| Gastric | 17 (1.0%) | 59 (1.0%) | 76 (1.0%) |
| Testicular | 9 (0.5%) | 53 (0.9%) | 62 (0.8%) |
| Small Intestine | 14 (0.9%) | 42 (0.7%) | 56 (0.8%) |
| Cervical | 8 (0.5%) | 41 (0.7%) | 49 (0.7%) |
| Peritoneal | 13 (0.8%) | 31 (0.5%) | 44 (0.6%) |
| Appendix | 6 (0.4%) | 34 (0.6%) | 40 (0.5%) |
| Esophageal | 10 (0.6%) | 30 (0.5%) | 40 (0.5%) |
| Fallopian | 6 (0.4%) | 25 (0.4%) | 31 (0.4%) |
| Larynx | 13 (0.8%) | 18 (0.3%) | 31 (0.4%) |
| Unknown | 2 (0.1%) | 25 (0.4%) | 27 (0.4%) |
| Genital Other | 5 (0.3%) | 21 (0.4%) | 26 (0.4%) |
| Rectosigmoid | 3 (0.2%) | 23 (0.4%) | 26 (0.4%) |
| Urothelial | 3 (0.2%) | 18 (0.3%) | 21 (0.3%) |
| Liver | 7 (0.4%) | 11 (0.2%) | 18 (0.2%) |
| Ocular | 2 (0.1%) | 13 (0.2%) | 15 (0.2%) |
| Pharynx | 3 (0.2%) | 12 (0.2%) | 15 (0.2%) |
| Anal | 2 (0.1%) | 8 (0.1%) | 10 (0.1%) |
| Cardiac | 5 (0.3%) | 4 (0.1%) | 9 (0.1%) |
| Adrenal | 4 (0.2%) | 4 (0.1%) | 8 (0.1%) |
| Bile duct | 2 (0.1%) | 6 (0.1%) | 8 (0.1%) |
| Mediastinal | 0 (0%) | 7 (0.1%) | 7 (0.1%) |
| Urinary | 4 (0.2%) | 2 (0.0%) | 6 (0.1%) |
| Digestive Other | 1 (0.1%) | 4 (0.1%) | 5 (0.1%) |
| Gallbladder | 2 (0.1%) | 2 (0.0%) | 4 (0.1%) |
| Vascular | 2 (0.1%) | 1 (0.0%) | 3 (0.0%) |
| Parathyroid | 0 (0%) | 1 (0.0%) | 1 (0.0%) |
| Blood | 0 (0%) | 0 (0%) | 0 (0%) |
| Bone marrow | 0 (0%) | 0 (0%) | 0 (0%) |
| Lymph | 0 (0%) | 0 (0%) | 0 (0%) |

*Patients with hematologic cancers were excluded due to hyperglycemia associated with corticosteroid treatment.*

**Supplementary Table 3**. Descriptive statistics in patients with diabetes prior to cancer diagnosis (N=1,008).

| **Characteristic** | **N (%) / Mean (SD)** |
| --- | --- |
| **Age (years)** | 63.5 (12.5) |
| **Sex** |  |
| Female | 510 (50.6%) |
| Male | 498 (49.4%) |
| **Race^a^** |  |
| White | 852 (84.5%) |
| Black | 11 (1.1%) |
| Asian | 18 (1.8%) |
| Native Hawaiian/PI | 13 (1.3%) |
| American Indian/Alaska Native | 28 (2.8%) |
| Multi-racial | 9 (0.9%) |
| Missing | 77 (7.6%) |
| **BMI^b^ (kg/m^2)** |  |
| Normal weight | 154 (15.3%) |
| Underweight | 9 (0.9%) |
| Overweight | 273 (27.1%) |
| Obese | 521 (51.7%) |
| Missing | 51 (5.1%) |
| **Smoking** |  |
| Never | 134 (13.3%) |
| Passive | 2 (0.2%) |
| Quit | 25 (2.5%) |
| Yes | 26 (2.6%) |
| Missing | 821 (81.4%) |
| **Cancer stage** |  |
| Stage 0 | 34 (3.4%) |
| Stage I | 282 (28.0%) |
| Stage II | 127 (12.6%) |
| Stage III | 115 (11.4%) |
| Stage IV | 161 (16.0%) |
| Missing | 289 (28.7%) |
| **Vital status** |  |
| Alive | 790 (78.4%) |
| Dead | 218 (21.6%) |
| **Corticosteroids** |  |
| None | 492 (48.8%) |
| Before/at cancer diagnosis | 109 (10.8%) |
| After cancer diagnosis | 285 (28.3%) |
| Before and after cancer diagnosis | 122 (12.1%) |
| **ORC** |  |
| No | 533 (52.9%) |
| Yes | 475 (47.1%) |
| **Prediabetes/diabetes type** |  |
| Drug induced | 1 (0.1%) |
| Hyperglycemia/prediabetes | 172 (17.1%) |
| Type 1 | 101 (10.0%) |
| Type 2 | 733 (72.7%) |
| **Years to diabetes diagnosis (years)** 3.72 (4.56) | |
| **Cancer treatment** |  |
| No treatment | 82 (8.1%) |
| Surgery only | 345 (34.2%) |
| Systemic treatment only | 158 (15.7%) |
| Other | 423 (42.0%) |
| *^a^PI = Pacific Islander.*  *^b^BMI (body mass index) closest to cancer diagnosis.* | |

**Supplementary Table 4:** Results for sensitivity analyses, including lag analysis, complete case analysis, and an analysis excluding pancreatic cancer.

|  | **N (%)** | **HR (95% CI)** |
| --- | --- | --- |
| **Lag analysis (6 months)** |  |  |
| No prediabetes/diabetes | 5655 (87) | REF |
| New-onset prediabetes/diabetes | 851 (13) | 3.06 (2.61 to 3.60) |
| **Complete case analysis** |  |  |
| No prediabetes/diabetes | 743 (77) | REF |
| New-onset prediabetes/diabetes | 228 (24) | 2.16 (1.63 to 2.87) |
| **Excluding pancreatic cancer** |  |  |
| No prediabetes/diabetes | 5478 (78) | REF |
| New-onset prediabetes/diabetes | 1561 (22) | 1.96 (1.73 to 2.22) |

*All models were adjusted for age, sex, race, cancer stage, cancer treatment type, corticosteroid use, smoking, body mass index (BMI) at cancer diagnosis, and presence of comorbidities.*

**Supplementary Figure 1**. Flow diagram of inclusion and exclusion criteria.
