# Supplementary figures and images for: New‐onset prediabetes/diabetes worsens overall survival in patients with cancer: A real‐world retrospective cohort study
Source: Diabetes Obes Metab. 2025 Nov 24;28(2):1247–57. doi: 10.1111/dom.70311 (PMC12649824; doi:10.1111/dom.70311)

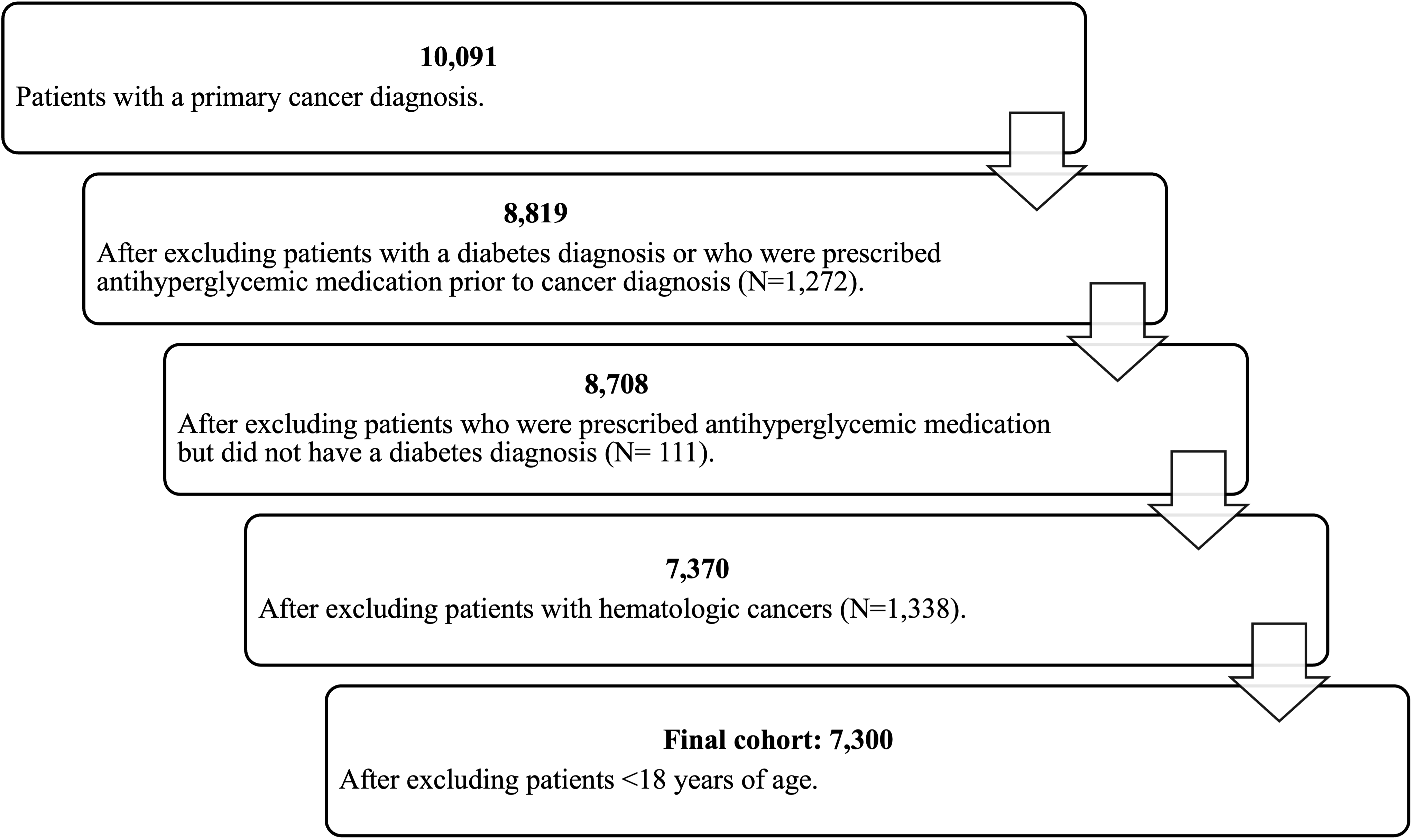

Supplement: Supplementary file 2 — Figure S1. Flow diagram of inclusion and exclusion criteria. [file DOM-28-1247-s002.png]
